# Supplementary material for: Intergenerational educational mobility in Bangladesh
Source: PLoS One. 2021 Jul 30;16(7):e0255426. doi: 10.1371/journal.pone.0255426 (PMC8323893; doi:10.1371/journal.pone.0255426)
Supplement: S1 Table — (DOCX) [file pone.0255426.s001.docx]

**S1 Table. State transition probability matrices for rural and urban areas.**

|  | **Rural** | **Urban** |
| --- | --- | --- |
| **Father to son or daughter** | \|  \| **1** \| **2** \| **3** \| **4** \| **5** \| \| --- \| --- \| --- \| --- \| --- \| --- \| \| **1** \| 0.41 \| 0.34 \| 0.22 \| 0.02 \| 0.01 \| \| **2** \| 0.14 \| 0.26 \| 0.51 \| 0.04 \| 0.05 \| \| **3** \| 0.11 \| 0.24 \| 0.44 \| 0.09 \| 0.12 \| \| **4** \| 0.03 \| 0.16 \| 0.45 \| 0.14 \| 0.22 \| \| **5** \| 0.02 \| 0.06 \| 0.33 \| 0.18 \| 0.41 \| | \|  \| **1** \| **2** \| **3** \| **4** \| **5** \| \| --- \| --- \| --- \| --- \| --- \| --- \| \| **1** \| 0.28 \| 0.36 \| 0.31 \| 0.03 \| 0.02 \| \| **2** \| 0.07 \| 0.22 \| 0.55 \| 0.10 \| 0.06 \| \| **3** \| 0.04 \| 0.14 \| 0.40 \| 0.18 \| 0.24 \| \| **4** \| 0.00 \| 0.03 \| 0.31 \| 0.15 \| 0.51 \| \| **5** \| 0.01 \| 0.01 \| 0.13 \| 0.17 \| 0.68 \| |
| **Father to Son** | \|  \| **1** \| **2** \| **3** \| **4** \| **5** \| \| --- \| --- \| --- \| --- \| --- \| --- \| \| **1** \| 0.36 \| 0.36 \| 0.22 \| 0.04 \| 0.02 \| \| **2** \| 0.07 \| 0.24 \| 0.54 \| 0.07 \| 0.08 \| \| **3** \| 0.08 \| 0.22 \| 0.39 \| 0.13 \| 0.18 \| \| **4** \| 0.03 \| 0.14 \| 0.47 \| 0.14 \| 0.22 \| \| **5** \| 0.00 \| 0.09 \| 0.27 \| 0.14 \| 0.50 \| | \|  \| **1** \| **2** \| **3** \| **4** \| **5** \| \| --- \| --- \| --- \| --- \| --- \| --- \| \| **1** \| 0.25 \| 0.36 \| 0.32 \| 0.04 \| 0.03 \| \| **2** \| 0.03 \| 0.23 \| 0.53 \| 0.12 \| 0.09 \| \| **3** \| 0.03 \| 0.11 \| 0.32 \| 0.19 \| 0.35 \| \| **4** \| 0.00 \| 0.05 \| 0.18 \| 0.17 \| 0.60 \| \| **5** \| 0.00 \| 0.01 \| 0.05 \| 0.16 \| 0.78 \| |
| **Father to Daughter** | \|  \| **1** \| **2** \| **3** \| **4** \| **5** \| \| --- \| --- \| --- \| --- \| --- \| --- \| \| **1** \| 0.45 \| 0.32 \| 0.21 \| 0.01 \| 0.01 \| \| **2** \| 0.20 \| 0.27 \| 0.49 \| 0.02 \| 0.02 \| \| **3** \| 0.14 \| 0.25 \| 0.48 \| 0.06 \| 0.07 \| \| **4** \| 0.03 \| 0.18 \| 0.42 \| 0.15 \| 0.22 \| \| **5** \| 0.04 \| 0.04 \| 0.37 \| 0.22 \| 0.33 \| | \|  \| **1** \| **2** \| **3** \| **4** \| **5** \| \| --- \| --- \| --- \| --- \| --- \| --- \| \| **1** \| 0.33 \| 0.35 \| 0.29 \| 0.02 \| 0.01 \| \| **2** \| 0.10 \| 0.22 \| 0.57 \| 0.07 \| 0.04 \| \| **3** \| 0.05 \| 0.17 \| 0.45 \| 0.17 \| 0.16 \| \| **4** \| 0.00 \| 0.01 \| 0.42 \| 0.14 \| 0.43 \| \| **5** \| 0.01 \| 0.01 \| 0.22 \| 0.18 \| 0.58 \| |

**Note:** 1=illiterate, 2=primary, 3=secondary, 4=higher secondary, and 5=higher education.
