# Supplementary material for: Intergenerational educational mobility in Bangladesh
Source: PLoS One. 2021 Jul 30;16(7):e0255426. doi: 10.1371/journal.pone.0255426 (PMC8323893; doi:10.1371/journal.pone.0255426)
Supplement: Attachment — Submitted filename: Reviewers Comments.pdf [file pone.0255426.s003.pdf]

Reviewer's Comments:

The authors of this paper have attempted to analyze the social mobility in Bangladesh using the Markov Chain model. Scientific research related to social mobility in Bangladesh using a probabilistic approach is hardly seen in the existing literature, and filling this gap is the novelty of this work. However, I have some important comments regarding their work which are mentioned below:

(i) This study considers that the socio-economic conditions of offspring depend only on their father's socio-economic conditions. But in reality, offspring socio-economic conditions depend not only on their father's socio-economic conditions but also mother socio-economic conditions, sibling's socio-economic conditions, gender, region and etc. Even this is evident in the descriptive statistics provided in Table 2. The authors may use multiple logistic regression as a very basic model before considering any sophisticated model which offers the flexibility to accommodate other covariates as well along with father's socio-economic conditions to model offspring socio-economic conditions.

(ii) Under the current Markov Chain framework where offspring socio-economic conditions depend only on their father's socio-economic conditions is treated as the simplest type of model to analyze social mobility (Borah. S (2013)). It would be really interesting work if authors can come up with new modelling tricks to model offspring socio-economic conditions which can take into account for other covariates as well.

Smita Borah, Stochastic Modelling of Social Mobility: A Case Study in Golaghat, Assam, *International Journal of Statistics and Applications,* Vol. 3 No. 3, 2013, pp. 43-49

(iii) In Table 3, authors present a transition probability matrix for "Father to All". But the term "Father to All" is not defined anywhere before, which may make the reader confused. I guess they intended to mean all the children of a father belong to the same class at time t. For example, suppose a father has 3 children. Then all the three children will achieve the same education level at time t. If this is the case then it oversimplifies the real situation. If this is not the case then the Markov Chain does not seem applicable here.

(iv) Transition probability matrix for "Father to Son" has valid probabilistic interpretation when a father has only one son. If a father has more than one son then the Markov Chain framework fails to model this situation because of not having a valid transition probability matrix. For example, suppose in class 1 there are 15 fathers i.e. $n_1 = 15$. When one father has more than one son then $\sum_{j=1}^{5} n_{1j} > 15$ which destroys the property of the transition probability matrix. Country like Bangladesh not having a stable population structure is very likely that every father has an average

of more than one son. Under this condition, considering the Markov Chain to model offspring socio-economic conditions is not valid.

(v) Comment mentioned in (iv) also applicable for the transition probability matrix for "Father to Daughter"

Finally, I would like to recommend this paper for possible publication to PLOS ONE **subject to the satisfactory major revision of the aforementioned points.**
