# Supplementary material for: Intergenerational educational mobility in Bangladesh
Source: PLoS One. 2021 Jul 30;16(7):e0255426. doi: 10.1371/journal.pone.0255426 (PMC8323893; doi:10.1371/journal.pone.0255426)
Supplement: Attachment — Submitted filename: Response to Reviewers.docx [file pone.0255426.s004.docx]

**Author responses to the review comments:**

We would like to express our sincere gratitude to the two anonymous reviewers and the Academic Editor for their valuable comments. We have considered all the comments made by the reviewers and thoroughly revised and formatted the manuscript accordingly. A detailed response to each of the comments is provided in the table below:

| **Academic Editor comments:** | **Response** | **Note** |
| --- | --- | --- |
| Please include the following items when submitting your revised manuscript:   - A rebuttal letter that responds to each point raised by the academic editor and reviewer(s). You should upload this letter as a separate file labeled 'Response to Reviewers'. - A marked-up copy of your manuscript that highlights changes made to the original version. You should upload this as a separate file labeled 'Revised Manuscript with Track Changes'. - An unmarked version of your revised paper without tracked changes. You should upload this as a separate file labeled 'Manuscript'.   If you would like to make changes to your financial disclosure, please include your updated statement in your cover letter. Guidelines for resubmitting your figure files are available below the reviewer comments at the end of this letter. | The required files are submitted through the submission system.  Not applicable. |  |
| Journal Requirements:  When submitting your revision, we need you to address these additional requirements.  Please ensure that your manuscript meets PLOS ONE's style requirements, including those for file naming. The PLOS ONE style templates can be found at  https://journals.plos.org/plosone/s/file?id=wjVg/PLOSOne_formatting_sample_main_body.pdf and  https://journals.plos.org/plosone/s/file?id=ba62/PLOSOne_formatting_sample_title_authors_affiliations.pdf | The manuscript is revised according to the PLOS ONE’s style. |  |
| 2. We note that you have indicated that data from this study are available upon request. PLOS only allows data to be available upon request if there are legal or ethical restrictions on sharing data publicly. For more information on unacceptable data access restrictions, please see http://journals.plos.org/plosone/s/data-availability#loc-unacceptable-data-access-restrictions.    In your revised cover letter, please address the following prompts:  2a) If there are ethical or legal restrictions on sharing a de-identified data set, please explain them in detail (e.g., data contain potentially sensitive information, data are owned by a third-party organization, etc.) and who has imposed them (e.g., an ethics committee). Please also provide contact information for a data access committee, ethics committee, or other institutional body to which data requests may be sent.  2b) If there are no restrictions, please upload the minimal anonymized data set necessary to replicate your study findings as either Supporting Information files or to a stable, public repository and provide us with the relevant URLs, DOIs, or accession numbers. For a list of acceptable repositories, please see http://journals.plos.org/plosone/s/data-availability#loc-recommended-repositories.  We will update your Data Availability statement on your behalf to reflect the information you provide. | All the data are contained in the manuscript in summarized form. The authors cannot share the full data set publicly because the data are owned by Research, Training and Management (RTM) International, and authors do not have permission to share the data.  But the whole data set may be accessed from RTM International on request. Email to Executive Director:  [jpasha@rtm-international.org](mailto:jpasha@rtm-international.org)  with copy to the corresponding author Mohammed Nazmul Huq: [nhuq@juniv.edu](mailto:nhuq@juniv.edu) |  |

| **Reviewer 1 comments:** | **Response** | **Note** |
| --- | --- | --- |
| The authors of this paper have attempted to analyze the social mobility in Bangladesh using the Markov Chain model. Scientific research related to social mobility in Bangladesh using a probabilistic approach is hardly seen in the existing literature, and filling this gap is the novelty of this work. However, I have some important comments regarding their work which are mentioned below:  (i) This study considers that the socio-economic conditions of offspring depend only on their father's socio-economic conditions. But in reality, offspring socio-economic conditions depend not only on their father's socio-economic conditions but also mother socio-economic conditions, sibling’s socio-economic conditions, gender, region and etc. Even this is evident in the descriptive statistics provided in Table 2. The authors may use multiple logistic regression as a very basic model before considering any sophisticated model which offers the flexibility to accommodate other covariates as well along with father's socio-economic conditions to model offspring socio-economic conditions. | We highly appreciate this comment.  We acknowledge the reviewer’s comment on the use of other different models for measuring intergenerational mobility, e.g. multiple logistic regression model. We also reviewed several modelling approaches to describe the intergenerational mobility status of the offspring using education, income and occupation. The use of other regression models (e.g. semi-log or log-linear model) is also common in such analysis. However, the use of such models requires access to the other relevant explanatory variables. Due to unavailability of the reliable explanatory variables, we could not adopt such regression models for the analysis. Further, the use of logistic regression can be useful to establish the relationship between the educational attainment of offspring and their fathers but may not be suitable for measuring intergenerational mobility.  Moreover, the mobility indices can also explain the dependence of offspring’s socio-economic status to their father’s socio-economic status. This is because that *the lower the mobility index, the higher the dependence of the socio-economic status of the offspring to their father’s socio-economic status*. In contrast, the higher mobility index represents lower dependence.  Thus, in this paper, we aimed to measure the intergenerational educational mobility by using only the stochastic modelling approach. | Revised texts are in red color.  Page: |
| (ii) Under the current Markov Chain framework where offspring socio-economic conditions depend only on their father's socio-economic conditions is treated as the simplest type of model to analyze social mobility (Borah. S (2013)). It would be really interesting work if authors can come up with new modelling tricks to model offspring socio-economic conditions which can take into account for other covariates as well.  Smita Borah, Stochastic Modelling of Social Mobility: A Case Study in Golaghat, Assam, International Journal of Statistics and Applications, Vol. 3 No. 3, 2013, pp. 43-49. | In the existing literature there is no study found to use stochastic modelling approach for finding the educational mobility in Bangladesh. This gap motivates us for doing this study and deciding to identify the contribution of education to their next generation.  Borah (2013) consider only occupational status in his model. However, we used “education” to measure the intergenerational mobility, which mainly considers the movement counts of the educational attainment of offspring (upward or downward) as compared to their father’s educational status (Table 2).  The authors strongly believe that a future study will be worthful to incorporate different covariates like, both father and mother’s education and occupation, income, sex, religion, etc. and to use regression models (e.g. multiple regression model) as well as stochastic modelling approach to make a comparative study.  We have added this as a limitation of the study and recommended a future study incorporating other regression models. | Revised texts are in red color.  Page: 17 |
| (iii) In Table 3, authors present a transition probability matrix for "Father to All". But the term "Father to All" is not defined anywhere before, which may make the reader confused. I guess they intended to mean all the children of a father belong to the same class at time t. For example, suppose a father has 3 children. Then all the three children will achieve the same education level at time t. If this is the case then it oversimplifies the real situation. If this is not the case then the Markov Chain does not seem applicable here. | The authors are grateful to the reviewer for pointing out this issue.  We have rephrased “Father to All” as “Father to son or daughter” in the revised manuscript.  In fact, we consider only one child for each father. | Revised texts are in red color. Page: 7, 9, 13, 14, 22 |
| (iv) Transition probability matrix for "Father to Son" has valid probabilistic interpretation when a father has only one son. If a father has more than one son then the Markov Chain framework fails to model this situation because of not having a valid transition probability matrix. For example, suppose in class 1 there are 15 fathers i.e. n_1=15. When one father has more than one son then ∑_(j=1)^5▒〖n_1j>15〗 which destroys the property of the transition probability matrix. Country like Bangladesh not having a stable population structure is very likely that every father has an average of more than one son. Under this condition, considering the Markov Chain to model offspring socio-economic conditions is not valid. | Thank you very much for pointed out this issue.  We agree with the statement that “Bangladesh not having a stable population structure is very likely that every father has an average of more than one son”. However, we considered only one child for each father, and used the stochastic modelling approach anticipating that our results apply in an average sense. | Revised texts are in red color.  Page: 7, 9 |
| (v) Comment mentioned in (iv) also applicable for the transition probability matrix for "Father to Daughter". | As described above, we only consider one child either a son or a daughter for each father. | Revised texts are in red color.  Page: 7, 9 |
| Finally, I would like to recommend this paper for possible publication to PLOS ONE subject to the satisfactory major revision of the aforementioned points. | Thanks for all the insightful comments of the reviewer, which help to improve our research. We have tried to incorporate the valuable comments of the reviewer into the revised manuscript. |  |

| **Reviewer 2 comments:** | **Response** | **Note** |
| --- | --- | --- |
| Upward social mobility, especially educational mobility, is an indication of improvement in a society. This nice work tries to evaluate intergenerational educational mobility in Bangladesh. The following suggestions will be helpful for improving the quality of the article.  1. Social mobility means mobility of different socio-economic factors across generations. This article deals with only one variable ‘educational status’. Here title is general but work is specific. Therefore, title should be specific like “Measuring Intergenerational Educational mobility in Bangladesh”. Again, Transition probability matrix (TPM) and Markov chain are well known stochastic approaches. So, name of the method should not mention in the title. It should be focused in methodology. | Thank you very much for your valuable comment and suggestions that help us to improve the quality of the manuscript.  We have revised the title as per suggestions of the reviewer. | Revised title is in red color.  Page: 1 |
| 2. ‘Educational mobility’ should be highlighted in the 'introduction' especially in the objective of the study. | The introduction section is updated as per comment. | Revised texts are in red color.  Page: 4 |
| 3. Rationale of the study should be clearly focused in the ending part of the “introduction”. | We appreciate the feedback. We have added the rationale in the last part of the introduction. | Revised texts are in red color. Page: 4, 5 |
| 4. In Markov chain, state j and k should be matched each other. In this study, State ‘j’ represents “Father Education level” and state ‘k’ represents “offspring, son or daughter’s education level”. Here, Matching between two states is little bit confusing. Author’s reference articles are related with the intergenerational social classes (lower class, middle class, upper class etc.) which are more general and matched with each other in broader perspectives. It is better to refer work on intergenerational social mobility, especially educational mobility, between parents and offspring. Followings are some observations regarding this issue:  (i) Here transitions are: Father to all, Father to son, and father to daughter. I think limiting probability is possible to interpret Father to son only. Here, interpretation of limiting probabilities is not clear enough. It will be better to consider transition:  (a) Father to son (potential father)  (b) Mother to daughter (potential mother)  And then interpret the limiting probabilities.  (ii) For a stochastic matrix row sum must be unity. Please check the row sums in the matrices. Some miscalculations are as follows:  (a) 1st matrix (Father to all), row-5 (Table 3).  (b) 2nd matrix (Father to son), row-1 (Table 3).  (c) 3rd matrix (Father to daughter), row-1 (Table 3). | In order to find the intergenerational mobility, we have considered ‘education’ and the states were defined as:   - Illiterate; - Completed primary education; - Completed secondary education; - Completed higher secondary; education; and - Completed graduation or post-graduation.   We used the same classification for both father and offspring, which matched each other.  We have added few references that describes intergenerational mobility measured in terms of ‘education’.  In this study, we consider only father’s educational status. The reason for not including mother’s educational status is that about three-fourth of the sampled mothers were illiterate and only one percent completed HSC or higher education levels. With such small number of mothers in the upper education categories, it was not possible to construct the Transition Probability Matrix for ‘mother to daughter’.  We strongly believe that it would be a potential factor to include mother’s educational status in future study with substantially large number of sample size to ensure adequate number of mothers in the higher education categories. We mention it in the limitation section of the study.  It happened due to rounding of the decimal places in Excel. Table 3 is corrected. | Revised texts are in red color.  Page: 4, 13, 14, 17, 22 |
| 5. Some points to be noted:  (a) In table 2, column-2 label ‘Sable’ should be ‘stable’. Please correct it.  (b) “An illiterate father is three times less likely to send their sons to schools as compared to their daughters” (abstract: line no. 39-40). Without logistic regression model this finding is confusing. Please check it.  (c) Please check grammar especially comparative degree throughout the manuscript and correct accordingly. | Thank you very much for carefully checking the manuscript.  The typo is corrected.  Abstract is revised.  All grammatical issues are fixed in the revised manuscript. | Revised texts are in red color.  Page: 2, 7, 12-13 |
|  |  |  |

The revised manuscript has been produced in accordance with the valuable comments and suggestions of the reviewers. Once again, we would like to thank the reviewers for their sincere dedication, professional insights, and earnest cooperation in reviewing the manuscript.
